# Metal artifact reduction by virtual monoenergetic reconstructions from spectral brain CT

**DOI:** 10.1016/j.ejro.2023.100479

**Published:** 2023-02-03

**Authors:** Helena Mellander, Veronica Fransson, Kristina Ydström, Jimmy Lätt, Teresa Ullberg, Johan Wassélius, Birgitta Ramgren

**Affiliations:** a Department of Medical Imaging and Physiology, Skåne University Hospital, Lund, Sweden; b Department of Clinical Sciences, Lund University, Lund, Sweden; c Radiation Physics, Department of Hematology, Oncology and Radiation Physics, Skåne University Hospital, Lund, Sweden

**Keywords:** CT, computed tomography, DECT, dual energy computed tomography, keV, kiloelectron volt, VMIs, virtual monoenergetic images, MRI, magnetic resonance imaging, DSA, digital subtraction angiography, CIs, conventional images, PACS, Picture Archiving and Communication System, DLP, dose length product, WM, white matter, ROI, region of interest, SD, standard deviation, HU, Hounsfield units, SNR, signal-to-noise ratio, IQR, interquartile range, CSF, cerebrospinal fluid, Diagnostic imaging, X-ray computed tomography, Metal artifacts, Monoenergetic imaging, Aneurysm

## Abstract

**Purpose:**

Conventional computed tomography (CT) images are severely affected by metal artifacts in patients with intracranial coils. Monoenergetic images have been suggested to reduce metal artifacts.

The aim of this study was to assess metal artifacts in virtual monoenergetic images (VMIs) reconstructed from spectral brain CT.

**Methods:**

Thirty-two consecutive patients with intracranial coils examined by spectral non contrast brain CT (NCCT) at our center between November 2017 and April 2019 were included. Attenuation and standard deviations were measured in regions of interest (ROIs) at predefined areas in artifact-free and artifact-affected areas. Measurements were performed in conventional polyenergetic images (CIs) and the corresponding data for VMIs were retrieved through spectral diagrams for the each ROI. Subjective analysis was performed by visual grading of CIs and specific VMIs by two neuroradiologists, independently.

**Results:**

In artefact-affected image areas distal from the metal objects, the attenuation values decreased with higher energy level VMIs. The same effect was not seen for artefact-affected image areas close to the metal.

Subjective rating of the artefact severity was significantly better in VMIs at 50 keV for one of the two reviewers compared to the CIs. Overall image quality and tissue differentiation scores were significantly higher for both reviewers in VMIs at 60 and 70 keV compared to CIs.

**Conclusion:**

Our quantitative and qualitative image analysis shown that there is a small significant reduction of intracranial coils artifacts severity by all monoenergetic reconstructions from 50 to 200 keV with preserved or increased overall subjective image quality compared to conventional images.

## 1 Introduction

Artifacts caused by metal implants are known to impair evaluation of brain computed tomography (CT). The artifacts are, in part, caused by beam hardening i.e. dense materials or tissues that selectively filter out low energy x-ray beams, resulting in distorted attenuation in the surrounding tissues [1], [2], [3]. Metal implants are not uncommon and are used in a variety of treatments for intracranial pathology, such as aneurysm occlusion and symptomatic treatment with deep brain stimulation-electrodes in patients with Parkinson’s disease [4], [5].

Unlike conventional polyenergetic CT, dual energy CT (DECT) enables viewing of virtual images at different kiloelectron volts (keVs). Dual energy imaging can be acquired by using different tube voltages, tubes that rapidly switch voltage or through a dual layer detector. The technique is usually manufacturer-dependent, but an in common benefit is the possibility of reconstructing virtual monoenergetic images (VMIs) at a spectrum of energy levels. Reconstructing VMIs at a high keV could decrease the amount of metal artifacts since the beam hardening effect is reduced, as shown in previous studies [1], [6]. Other potential advantages of DECT over conventional CT include improved tissue differentiation at low keVs, and the possibility of reconstructing virtual non contrast (VNC) images [7], [8], [9], [10], [11].

Patients with intracranial metal implants are often followed up with CT imaging after receiving the implant but may also be referred for other reasons. Reduced metal artifacts on CT images is therefore a major clinical need.

The aim of this study was to assess metal artifacts in virtual monoenergetic images (VMIs) reconstructed from spectral brain CT.

## 2 Material and methods

### 2.1 Study population

We performed a retrospective search in our Picture Archiving and Communication System (PACS) for patients who had undergone non-contrast brain CT in the IQon Spectral CT (Philips Healthcare Inc., The Netherlands) at our institute between November 21, 2017 and April 4, 2019. All patients aged 18 or more with intracranial coils were included. If a patient had multiple exams that met our inclusion criteria, the one performed closest to the time of the endovascular intervention was chosen.

Examinations lacking spectral images in PACS were excluded.

The study was approved by the Swedish Ethical Review Authority (reference number 2019–02225) and individual informed consent was waived.

### 2.2 CT parameters

If a patient had been treated with coils in more than one location, the largest skein of coils was used for measurements, and the measurements were done in images with artifacts from that skein of coils only.

Dose length product (DLP) was noted and the effective dose was calculated as *DLP*[mGycm]*0.0024[mSv/mGycm] [12].

Measurements to calculate the coil volume were obtained from 3D DSA images and calculated as 4/3$\pi r^{3}$, using the largest diameter to calculate the radius [13].

All images were assessed in the axial plane of 4 mm slice thickness images, in line with local routine.

### 2.3 Quantitative image analysis

Quantitative image analysis was based on measurements in predefined regions of interest (ROIs) manually drawn at specific locations in the CT images with conventional reconstruction (CI);.

1) artifact-free white matter (WM) between vertex and the lateral ventricle in either hemisphere;
2) artifact-free cerebrospinal fluid (CSF) in the left lateral ventricle;
3) artifact-affected distal white matter (≥2 cm from the coils);
4) artifact-affected proximal white matter (<2 cm from the coils).

All ROIs were of circular shape and drawn to a diameter of 10 ± 1 mm. ROI size was only adjusted when necessary to avoid adjacent tissue, for example in slim lateral ventricles. All measurements were performed in ISP (IntelliSpace Portal, version 10.1.4.21403, Philips healthcare Inc., The Netherlands).

The mean attenuation and the standard deviation (SD) measured in Hounsfield units (HU) were noted for each ROI in the CIs and, for the same ROIs, retrieved through spectral diagrams for monoenergetic reconstructions ranging from 40 to 200 keV with 10 keV increments.

### 2.4 Qualitative analysis

Qualitative analysis was performed by visual grading of the conventional image series and monoenergetic reconstructions at 40, 50, 60, 70, 80, 120, 140 and 200 keV, by two independent reviewers, both senior interventional neuroradiologists with over 10 years’ experience (BR and JW). Chosen keV levels that were included in this part of the analysis were based on the attenuation values from the quantitative data that indicated large changes mainly between the lower keV reconstructions, and from previous work [6], [7], [9]. Prior to the visual grading, example cases were used in order to establish the grading scale to the reviewers.

All images used for the qualitative analysis were de-identified and presented to the reviewers in random order without any patient- or reconstruction information using the Viewdex software version 2.272 (Viewer for Digital Evaluation of X-ray images) [14], [15]. The images were displayed on an image workstation with dedicated monitors for diagnostic radiology (Coronis® Fusion MDCC-6430 6MP, Barco, Kortrijk, Belgium). The contrast/brightness levels could be adjusted by the reviewer. All images were assessed on a 5-point Likert scale (1 =Non diagnostic/massive artifacts, 2 = Poor/severe artifacts 3 =Fair/moderate artifacts, 4 = Good/minor artifacts and 5 = Excellent/no artifacts) in coherence with previous work [7], [16], [17] regarding: 1) overall image quality, 2) presence of metal artifacts and 3) grey-white matter differentiation in artifact-free tissue.

Statistical analysis All analyses were performed using IBM SPSS Statistics version 25 (IBM Corp, Armonk, New York, USA). Descriptive data are presented as means and/or medians. A Wilcoxon signed-rank test was used for pairwise comparison of attenuation between specific VMIs. The level of statistical significance were predefined to *p* ≤ 0.05.

**Fig. 1:**
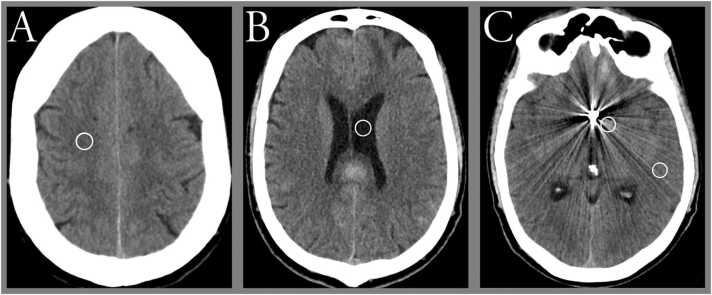
Example of placement of region of interest (ROI) circles; a) non-artifact white matter (ROI 1) between vertex and the lateral ventricle in either hemisphere, b) artifact-free cerebrospinal fluid (ROI 2) in the lateral ventricle (c), artifact-affected white matter distal (ROI 3) and proximal (ROI 4) to the metal implant.

Interobserver agreement was calculated as a weighted Cohens kappa coefficient using MedCalc Statistical Software version 20.009 (MedCalc Software, Ostend, Belgium) and Wilcoxon signed-rank test was used for comparison of scores between different reconstructions (Fig. 1).

## 3 Results

### 3.1 Patient population and coil parameters

The final study population was 32 patients, 19 women and 13 men. The mean age was 58 years. The most common coiling location was anterior communicating artery aneurysms, in 16 of the patients. The remaining coiling locations were the internal carotid artery, basilar artery, posterior inferior cerebellar artery, pericallosal artery, and vertebral artery. Baseline and coil parameters are shown in Table 1.

**Table 1:** Patient characteristics, CT scanning parameters and coil parameters for the study population.

Patient characteristics	
Number of patients	32
Men/women	13/19
Mean age (range) [years]	58 (25–84)
Subarachnoid hemorrhage	28 (88%)
CT scanning parameters	
Tube voltage [kV]	120
Collimation [mm]	64 × 0.625
Rotation time [s]	0.33
Pitch	0.359
Median CTDI_vol_ (IQR) [mGy]	40.3 (37–43)
Median DLP (IQR) [mGycm]	833 (744–891)
Median effective dose (IQR) [mSv]	2.0 (1.8–2.1)
Coil parameters	
Anterior communicating artery	16 (50%)
Internal carotid artery	9 (28%)
Basilar artery	2 (6%)
Other locations	5 (16%)
Median coil volume (IQR) [mm^3^]	93 (34–296)
n = number, mSv = milliSievert, IQR = Inter Quartile Range	

### 3.2 Quantitative image analysis

**Fig. 2:**
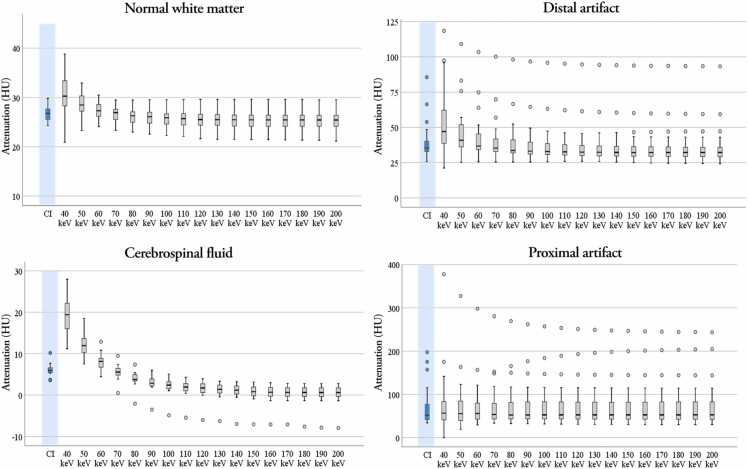
Box plots of attenuation values (Hounsfield units) from all measured ROIs in conventional images (CI, blue) and monoenergetic reconstructions from 40 to 200 keV (grey).

The attenuation values generally decreased with higher keV in ROIs 1–3. Box plots of HU values of artifact-free white matter, CSF and artifact-affected tissue distal and proximal to the metal implant are shown in Fig. 2.

Pairwise comparison of HU values was performed between CI and VMIs at 50, 60 and 70 keV, based on the results of the qualitative analysis. The tests showed significantly higher attenuation values in VMIs at 50 and 60 keV and non-significantly higher in VMIs at 70 keV reconstructions compared to CI for ROI 1 (artifact-free white matter).

For ROI 2 (CSF) the attenuation was significantly higher in 50 and 60 keV VMIs and significantly lower in 70 keV reconstructions compared to CIs. For ROI 3 (distal artifact) there were significant differences between CI and VMIs at 50 and 60 keV, with higher values in the VMIs, but no significant difference between CIs and 70 keV was found. No significant differences between CIs or the VMIs listed above were found for ROI 4 (proximal artifact).

### 3.3 Qualitative image analysis

**Fig. 3:**
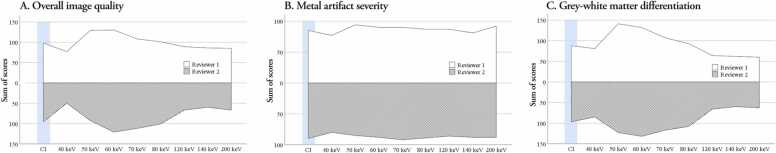
Area graphs representing sum of subjective image scores of both reviewers for the three items in the qualitative image analysis (**Overall image quality**, **Metal artifact severity** and **Grey-white matter differentiation**) for conventional images (CI, highlighted in light blue) and monoenergetic reconstructions at 40, 50, 60, 70, 80, 120, 140 and 200 keV. For all three items a higher score represent a better result, including **Metal artifact severity** where a higher score represent less disturbance from metal artifacts.

The results of overall image quality, metal artifact severity and grey-white matter differentiation scores for both reviewers are shown in Fig. 3. Based on visual assessment of the graphs, significance was tested between CIs and VMIs at 50, 60 and 70 keV.

**Fig. 4:**
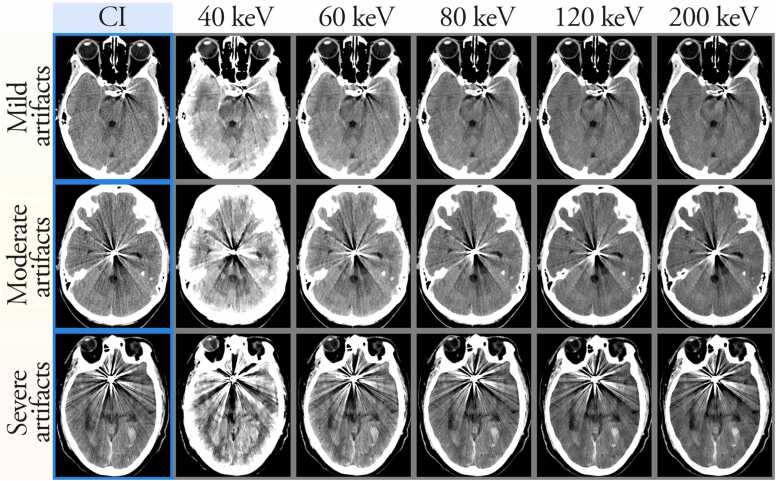
Example of conventional polyenergetic images (CI, left column highlighted in blue), followed by VMIs at 40, 60, 80 and 120 and 200 keV in typical examinations with mild (**top row**), moderate (**middle row**) and severe coil artifacts (**bottom row**).

Typical image examples for CIs and VMIs at 50, 60, 70 and 120 keV are shown in Fig. 4.

### 3.4 Overall image quality

The median image quality score peaked at 4.0 for both reviewers (in 50 and 60 keV reconstructions for Reviewer 1, and in 60 keV for Reviewer 2). The median score for CIs was 3.0 for both reviewers. The scores for overall image quality were significantly higher for VMIs at 50, 60 and 70 keV for Reviewer 1, and for VMIs at 60 and 70 keV for Reviewer 2 compared to CIs. The inter-observer agreement was fair (Cohens weighted kappa coefficient = 0.3).

### 3.5 Artifact Severity

The median artifact severity score was 3.0 for all VMIs except for the 40 keV reconstructions (scores of 2.5, and 2 for Reviewers 1 and 2, respectively). The median score for CIs was 2.5 for Reviewer 1 and 3.0 for Reviewer 2. The scores were significantly higher in VMIs at 50 keV than for CIs for Reviewer 1, but not for any of the VMIs compared to CI for Reviewer 2. The interobserver agreement was moderate (Cohens weighted kappa coefficient = 0.6).

### 3.6 Grey-white matter differentiation

The median grey-white matter differentiation score peaked at 4.5 in VMIs at 50 keV for Reviewer 1, and at 4.0 in VMIs at 50, 60 and 70 keV for Reviewer 2. The scores for VMIs at 50, 60 and 70 keV were all significantly higher than for the CIs for both reviewers.

The interobserver agreement was moderate (Cohens weighted kappa coefficient = 0.5).

## 4 Discussion

The aim of this study was to assess metal artifacts in virtual monoenergetic images (VMIs) reconstructed from spectral brain CT.

In our quantitative image analysis, we found that in artifact-affected image areas ≥ 2 cm distal from the coils, the attenuation values decreased with higher energy level VMIs. This is consistent with the quantitative image analysis that show improved overall image quality, improved grey-white matter differentiation and reduced metal artifact severity in VMIs at lower energy levels compared to CIs.

The same benefit of VMIs is not seen in artifact-affected image areas within 2 cm of the coils. A likely explanation is that photon starvation is the main cause of the proximal artifacts, whereas the beam hardening effect, that the dual energy CT technique mainly reduces, is more important further distal to the coils.

In a phantom study, Winklhofer et al. [6] evaluated artifacts produced by clips and coils of various volumes and densities in VMIs at 120 keV in combination with the iMAR metal artifact reduction algorithm and found that VMIs in combination with the iterative algorithm resulted in the least severe artifact scores, but this improvement was small for large and densely packed coils which could possibly be due to the predominance of photon starvation adjacent to dense metal. Große Hokamp et al. [18] showed that the metal artifacts from deep brain electrodes was reduced by using VMIs in combination with the O-MAR iterative metal artifact reduction algorithm. The results of these studies regarding VMIs are in line with ours.

The effect of metal artifact reduction achieved by dedicated post-processing algorithms [6], [18] seem to be more prominent than the effect of VMIs, but this may differ for metal artifacts generated by many small objects, such as the Tantalum in ONYX® or Squid®-embolization used for dural arteriovenous fistulas (DAVF) or chronic subdural hematomas (CSDH) instead of a single large metal object. Therefore, the use of VMIs as a complimentary measure to reduce metal artifacts may be of greater importance in such cases.

Other studies have shown significant metal artifact reduction in VMIs for various types of orthopedic implants [19], [20], [21]. Although not directly comparable, the effect on VMIs on intracranial metal artifacts seem to be less pronounced for intracranial coils than for orthopedic implants, this may be due to the surrounding cranium that possibly absorbs the higher energy beams to a great extent, or by the different physical properties of metal implants. For example, platinum used in neurovascular coils has an approximately five-fold density compared to titanium used in orthopedic implants [22], [23], which could affect the severity of metal artifacts.

The artifacts produced by coils are often heterogenic with alternating hypo- and hyperdense streaks, due to irregularities in aneurysm shape and coil packing density within the aneurysm [24]. In quantitative measurements, the hypo- and hyperattenuating streaks could possibly cancel each other out, and thus it is important to combine quantitative and qualitative evaluation of artifact severity. In our study, the quantitative and qualitative image evaluations are consistent and in support of each other.

A benefit of metal artifact reduction by VMIs is that it does not increase the patient’s radiation dose, which would typically be necessary for adjustments of scanning parameters to decrease metal artifacts, such as increasing the x-ray tube voltage. In addition, VMI reconstructions is rapid and easily accessible in the clinical day-to-day routine post-processing work, for examples if the presence of coils was not known beforehand.

Since we find that the reduction of metal artifact severity is relatively constant across all VMIs compared to CI we may also conclude that any other benefits of VMIs, such as increased diagnostic ability for early ischemia [25] may be utilized without aggravating any metal artifacts.

### 4.1 Limitations

This study has several limitations, the first being a relatively small study population, however many of the results was still shown to be statistically significant. The second limitation is that all data collection was done on the same CT scanner and all image processing was done on only one vendor-specific software. In future studies it would be valuable to include multiple scanners capable of generating a spectrum of VMIs from 40 to 200 keV. The third limitation is that we only included cases where the metal artifacts were caused by neurovascular coils. In future studies it would be valuable to evaluate artifacts from other intracranial implants such as clips and electrodes as well as artifacts from multiple locations such as Tantalum in CSDH embolization.

## 5 Conclusions

Our quantitative and qualitative image analysis shown that there is a small significant reduction of intracranial coils artifacts severity by monoenergetic reconstructions with preserved or increased overall subjective image quality compared to conventional images.

None of the monoenergetic reconstructions from 50 to 200 keV increased the severity of intracranial coils artifacts compared to conventional images.

Further studies, should also address the diagnostic ability with VMIs with and without iterative metal artifact reduction algorithms in assessing brain CT with metal artifacts, such as the ability to assess subarachnoid hemorrhage or ischemia.

## Ethics approval details

This study was approved and individual informed consent was waived by the Swedish Ethical Review Authority (reference number 2019–02225).

## Fundings

This study was funded by regional 10.13039/100001424ALF grants to HM, TU and JW, the 10.13039/501100003173Crafoord Foundation to JW and by SUS Stiftelser & Fonder to JW. None of the funders had any involvement in the planning of methodology, data retrieval or analysis in the study.

## CRediT authorship contribution statement

HM, VF, KY, TU, JW and BR conceptualized and planned the study. HM and VF collected data. HM, VF, JL and TU processed and analyzed data. HM wrote the first manuscript draft. All authors revised the manuscript. All authors have read and approved the final version of the manuscript.

## Conflicts of interest

None of the authors have any conflicts of interests related to the study. JW is a founder and shareholder of Uman Sense AB and has received speaker honoraria from Siemens Healthineers, BALT group and Medtronic Inc.

## Data Availability

An anonymized dataset supporting the conclusions of this article may be upon reasonable request including the appropriate ethics approval.
